# Rescue Inhaler Overuse in Severe Asthma: A Real-World Study of Short-Acting β_2_-Agonist and Short-Acting Muscarinic Antagonist Use

**DOI:** 10.3390/biomedicines14061332

**Published:** 2026-06-12

**Authors:** Elena Villamañán, Daniel Laorden, Carlos Carpio, Javier Domínguez-Ortega, Leticia De las Vecillas, David Romero, Diana Betancor, Carolina Alfonso, Susana De Andrés, Sonia Mallón, Eva Villarroya, Alejandro Soto, Carmen Sobrino, Marta Moro, Pablo Mariscal, Alicia Herrero, Santiago Quirce, Rodolfo Álvarez-Sala

**Affiliations:** 1 Pharmacy Department, Hospital Universitario La Paz, 28046 Madrid, Spain; elena.villamanan@salud.madrid.org (E.V.);; 2 Department of Medicine, Universidad Autónoma de Madrid, 28047 Madrid, Spain; carlosjavier.carpio@salud.madrid.org (C.C.);; 3 La Paz University Hospital Research Institute (IdiPAZ), 28046 Madrid, Spain; javier.dominguez@salud.madrid.org (J.D.-O.);; 4 Pulmonology Department, Hospital Universitario La Paz, 28046 Madrid, Spain; 5 Allergy Department, Hospital Universitario La Paz, 28046 Madrid, Spain; 6 Otorhinolaryngology Department, Hospital Universitario La Paz, 28046 Madrid, Spain

**Keywords:** severe asthma, overuse, rescue inhalers, biologics

## Abstract

**Background**: Overuse of short-acting reliever inhalers, particularly short-acting β_2_-agonists (SABAs), is linked to poor asthma control, higher exacerbation risk, and increased mortality. Data on reliever overuse in severe asthma and the role of short-acting muscarinic antagonists (SAMAs) remain limited. **Objective**: This study aims to assess the prevalence of rescue inhaler overuse in adults with severe asthma and examine its associations with maintenance therapy adherence, biologic treatment, oral corticosteroid use, asthma control, lung function, allergic sensitization, and comorbidities. **Methods**: We conducted a retrospective observational study of 223 adults with severe asthma followed at a tertiary multidisciplinary clinic in 2024. Clinical, functional, pharmacological, and pharmacy-dispensing records were reviewed. Rescue inhaler overuse was defined as dispensing ≥ 3 SABA and/or SAMA canisters per 12 months. Associations with clinical and treatment variables were analyzed. **Results**: Among 223 patients, 144 (64.6%) had a prescribed rescue inhaler; 85 (38.1%) met the criteria for overuse. Of those prescribed rescue therapy, 59.0% overused, with 47.2% classified as low overuse and 11.1% as high overuse. Overuse was more frequent in patients with maintenance adherence > 50%. Biologic therapy was associated with reduced odds of overuse, while oral corticosteroid use showed no significant effect. Poor asthma control (ACT < 20) was strongly associated with overuse. Lung function did not differ significantly, though overusers tended to have lower FEV_1_. Allergic sensitization was not associated with overuse; bronchiectasis was the comorbidity most frequently linked. **Conclusions**: Rescue inhaler overuse is common in severe asthma and closely linked to inadequate disease control. Lower overuse rates among biologic-treated patients suggest improved control reduces reliance on short-acting relievers. Systematic monitoring of reliever use may help identify high-risk patients and guide individualized management strategies.

## 1. Introduction

Asthma is a chronic inflammatory airway disease affecting millions worldwide, and its management requires strict symptom control and reduction in exacerbation risk through maintenance anti-inflammatory therapy and rescue bronchodilators [1,2]. Rescue inhalers are commonly used for rapid relief of symptoms [3,4,5,6,7].

In Spain, the SABINA study reported 28.7% of asthma patients overusing SABAs (≥3 inhalers/year), while 13.4% underused inhaled corticosteroids (≤4 inhalers/year) [8,9].

Overuse of rescue treatments is associated with an increased number of annual exacerbations and higher mortality rates [10,11,12,13,14,15]. In this context, the recent literature has called for a critical reappraisal of these therapies, highlighting the need for more cautious and monitored use in contemporary clinical practice, particularly given the availability of more effective anti-inflammatory strategies [16]. Therefore, systematic monitoring of rescue inhaler use has emerged as a key component in the management of asthma, especially in patients with severe disease.

This is often associated with underuse of inhaled corticosteroids, contributing to poor asthma control [17]. Current guidelines recommend Maintenance and Reliever Therapy (MART), combining inhaled corticosteroids with formoterol or SABAs, to improve disease management [3,18]. Since 2019, GINA no longer endorses SABA monotherapy for mild asthma, instead recommending inhaled corticosteroid–formoterol as anti-inflammatory reliever therapy for steps 1–2 and as maintenance plus MART for steps 3–5, given its superior reduction in severe exacerbations and hospital admissions [19]. This approach enables appropriate inhaler use through fixed daily dosing and reliever flexibility [20,21,22,23].

While SABA overuse is well documented in mild-to-moderate asthma, its use and potential overuse in severe asthma remain underexplored. Characterizing rescue inhaler patterns in this high-risk population is crucial to optimize management, reduce exacerbations, and improve quality of life. The present study evaluates rescue inhaler overuse in SA and examines whether patterns are influenced by pharmacological and clinical factors including lung function, comorbidities, and allergic status.

## 2. Methods

Retrospective observational study including adults with SA followed at the Severe Asthma Clinic (SAC) of a tertiary hospital. Routinely collected clinical data and community and hospital pharmacy dispensing records (January–December 2024) were used to evaluate treatment patterns in the full cohort of SA patients under active follow-up. All of them had uncontrolled asthma despite high-dose inhaled corticosteroids (ICS) combined with long-acting β_2_-agonists (LABA) and oral corticosteroids, according to contemporary guideline recommendations.

Eligible participants were aged ≥ 18 years, of either sex. Patients were excluded if pharmacy dispensing data were missing. Severe asthma was defined as disease that remained uncontrolled despite high-dose ICS/LABA therapy or required maintenance oral corticosteroids to achieve control, consistent with GINA Step 5 and GEMA Step 6 criteria [3].

### 2.1. Study Variables

Primary outcome: Number of patients overusing rescue inhalers based on medication dispensing records.

### 2.2. Overuse Assessment

Reliever overuse is currently defined as the use of ≥3 SABA inhalers/year, based on evidence from the SABINA programme [10,12,17,24].

Among reliever overusers, two categories were defined: high overusers (≥3 canisters every four months) and non-high overusers. Secondary outcomes included sociodemographic variables (age, sex) and pharmacological characteristics (maintenance inhaler therapy and adherence, biologic treatment, and systemic corticosteroid use).

Adherence to maintenance inhaler therapy was estimated using the medication possession ratio (MPR) calculated from pharmacy dispensing records. An MPR threshold > 50% was used to define acceptable adherence, reflecting real-world adherence patterns commonly reported among patients with asthma. Prescription and pharmacy refill data from the previous six months were analyzed to calculate adherence [23,24,25,26,27,28,29,30,31,32,33,34,35,36,37].

Additional secondary outcomes included clinical and laboratory parameters: asthma control (ACT), pulmonary function data (FEV_1_, FVC, FEV_1_/FVC and VC) and FeNO were retrieved from the clinic database. Spirometry was performed using a standardized laboratory spirometer, and percent predicted values for FEV_1_ and FVC were calculated using the Global Lung Function Initiative 2012 reference equations (GLI-2012) allergic sensitization, and asthma-related comorbidities [38]. Data were obtained from electronic medical records, SAC database, the centralized electronic prescribing system (MUP), and hospital pharmacy dispensing records.

### 2.3. Statistical Analysis

A study database was created with predefined ranges and consistency checks, and data quality was assessed through exploratory analyses to identify discrepancies and missing values. Continuous variables were summarized as means and standard deviations, and categorical variables as absolute and relative frequencies. Normality was assessed using the Shapiro–Wilk test. Group comparisons employed chi-square or Fisher’s exact tests, with odds ratios and 95% confidence intervals. Statistical analyses were performed using SAS 9.1 and SPSS 23.0.

### 2.4. Ethical Considerations

The Ethics Committee of Hospital Universitario La Paz approved the study (PI-6662) (14 May 2025). It was conducted in accordance with the Declaration of Helsinki. Patient data were anonymized, and confidentiality was ensured.

## 3. Results

Data from 223 severe asthma patients (mean age 61.3 ± 15.0 years; 70.4% female) were analyzed. Comorbidities were common, with gastroesophageal reflux (35.4%) and bronchiectasis (34.5%) most prevalent (Table 1).

Among the 223 patients analyzed, 64.6% (144/223) received a prescription for rescue inhalers (SABA/SAMAs). Reliever overuse was observed in 38.1% (85/223) of the cohort. The prevalence of overuse was lower in patients prescribed SAMAs compared with those prescribed SABAs (47.6% [10/21] vs. 61.0% [75/123]), although this difference did not reach statistical significance (OR 0.58; 95% CI 0.23–1.47; *p* = 0.337) (Table 2).

## 4. Rescue Overuse According to Pharmacological Outcomes

### 4.1. Maintenance Inhaled Therapy

**Figure 1:**
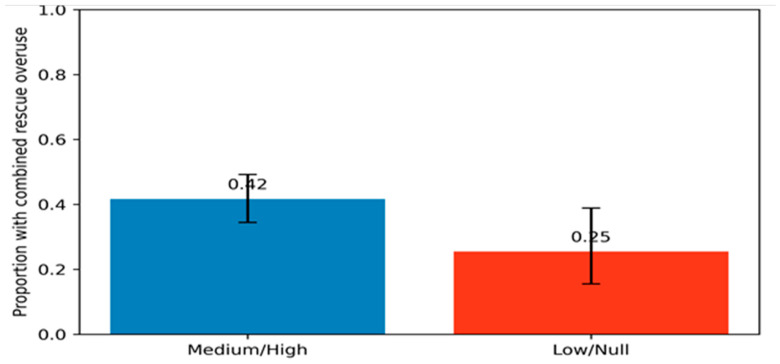
Overuse of rescue inhalers (SABAs plus SAMAs) in relation to adherence to maintenance inhaler therapy.

In this cohort, aggregated rescue inhaler overuse (SABA/SAMAs) was more frequent among patients with medium or high adherence (>50%) to maintenance therapy than among those with low or no adherence (<50%) (41.7% vs. 25.5%) (Figure 1).

### 4.2. Biologic Therapies

**Figure 2:**
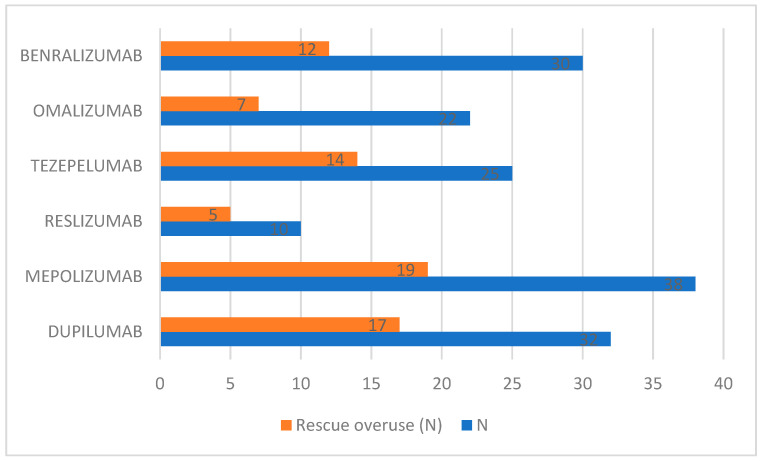
Overuse of rescue inhalers among patients receiving biologic therapy.

Of the 223 patients evaluated, 48.9% (109/223) were receiving biologic therapy for severe asthma. Reliever overuse was less frequent among patients on biologics compared with those not receiving biologic treatment (29.4% [32/109] vs. 45.6% [52/114]). Biologic therapy was associated with a significantly reduced risk of rescue medication overuse (OR 0.50; 95% CI 0.29–0.86; *p* = 0.018) (Figure 2).

### 4.3. Oral Corticosteroids

Rescue inhaler overuse did not differ significantly by concomitant OCS therapy. Although overuse was somewhat higher among OCS users (66.7% vs. 49.1%), the association was not statistically significant (OR 2.07; *p* = 0.277).

## 5. Rescue Overuse According to Clinical Outcomes

### 5.1. Asthma Control Test

Among 109 patients with available ACT and reliever data, rescue overuse was markedly more frequent in those with ACT < 20 compared with those ≥20 (74.4% vs. 47.0%). Patients with poor control had a significantly higher likelihood of overuse (OR 3.28; 95% CI 1.42–7.60; *p* = 0.0083).

In the multivariate analysis, which included all variables that were significant in the univariate models, poor asthma control (ACT < 20) remained an independent predictor of rescue inhaler overuse. Patients with inadequate control showed a markedly higher likelihood of overuse, with ACT ≥ 20 being associated with a 77% reduction in the odds of excessive reliever use (OR 0.23; 95% CI 0.10–0.55; *p* = 0.001) (Table 3).

### 5.2. Pulmonary Function

The association between pulmonary function parameters and reliever overuse was examined. Mean spirometric values (±SD)—including FEV_1_, FVC, FEV_1_/FVC ratio, VC, and fractional exhaled nitric oxide (FENO)—are presented in Table 3 according to SABA overuse status. Although patients with reliever overuse showed slightly lower mean lung function values, none of the differences were statistically significant (Table 4).

### 5.3. Allergies

Regarding allergen sensitization, 56.2% (81/144) of patients had documented allergies to dust mites, pollen, animal dander, or mold. Among sensitized patients, 51.8% (42/81) were classified as reliever overusers, compared with 66.7% (42/63) of non-allergic patients. This difference did not reach statistical significance (*p* = 0.062).

### 5.4. Asthma-Related Comorbidity

Finally, rescue inhaler overuse was examined according to asthma-related comorbidities. Patients with severe asthma and concomitant bronchiectasis showed the highest frequency of reliever overuse compared with the other comorbid conditions assessed (Table 5).

## 6. Discussion

In this severe asthma cohort, 64.6% of patients were prescribed rescue inhalers, and 38.1% met the criteria for overuse. This prevalence is higher than that reported in the Spanish SABINA study (28.7%), consistent with the well-described increase in SABA overuse among patients with more severe disease. The elevated rates observed likely reflect the greater symptom burden, clinical instability, and therapeutic complexity characteristic of severe asthma [17,24]. Importantly, our findings extend prior population-based observations by focusing specifically on a well-characterized severe asthma population, in whom treatment intensity and comorbidity burden are substantially higher.

The high prevalence of rescue inhaler overuse observed in our cohort should also be interpreted in light of recent evidence demonstrating that excessive short-acting β_2_-agonist (SABA) use is not merely a marker of poor disease control but is independently associated with adverse clinical outcomes. A recent systematic review and meta-analysis by Tsao et al. [5] showed that SABA overuse is associated with increased risks of severe exacerbations, hospitalizations, and mortality. These findings reinforce the clinical relevance of our results and position reliever overuse as a key indicator of disease instability and future risk. Importantly, this evidence supports a paradigm shift in asthma management, where SABA use is no longer considered benign but rather a modifiable risk factor.

In our study, reliever overuse was strongly associated with poor asthma control, as reflected by significantly higher odds among patients with ACT scores < 20. Our findings highlight ACT-defined asthma control as a key determinant of inappropriate reliever consumption in patients with severe asthma. This observation is consistent with the dose–response relationship described by Tsao et al. [5], whereby increasing SABA exposure is associated with progressively worse clinical outcomes. Notably, most cases in our cohort corresponded to low-level overuse, suggesting that even moderate excess use may carry clinically meaningful risk. This supports the concept of a continuum of risk rather than a clearly defined safety threshold, highlighting the importance of early identification and intervention even at relatively modest levels of overuse.

Several pathophysiological mechanisms may underlie the association between SABA overuse and adverse outcomes. Chronic exposure to β_2_-agonists may lead to receptor desensitization and downregulation, resulting in reduced bronchodilator responsiveness over time. In parallel, reliance on bronchodilation without concomitant anti-inflammatory therapy may allow persistent airway inflammation to remain untreated. As highlighted by Tsao et al. [5], this imbalance may contribute to worsening airway hyperresponsiveness, increased exacerbation risk, and potential disease progression. Additionally, frequent SABA use may mask symptom perception, leading to delayed medical review and underestimation of disease severity. These mechanisms are particularly relevant in severe asthma, where inflammatory pathways are often more complex and less responsive to standard therapies.

The absence of a significant association between reliever overuse and pulmonary function parameters in our study further supports the notion that spirometry alone does not adequately reflect disease activity or instability in severe asthma. This finding aligns with previous reports showing discordance between lung function and symptom burden or exacerbation risk. In this context, rescue inhaler use may represent a more sensitive and dynamic marker of real-world disease control, capturing fluctuations in symptoms that are not detected by periodic spirometric assessment.

The relationship between maintenance therapy adherence and reliever overuse was not straightforward. An additional consideration is the adherence threshold used in this study. Acceptable adherence was defined as an MPR > 50%, a cutoff selected to reflect real-world adherence patterns commonly observed in asthma populations. However, this threshold is lower than the ≥80% criterion frequently used in chronic disease research and may have reduced the contrast between adherent and non-adherent patients. Consequently, the observed association between maintenance therapy adherence and reliever overuse may have been attenuated. Future studies using more stringent adherence definitions could help clarify the extent to which inadequate maintenance treatment contributes to excessive reliance on rescue medication.

Higher overuse among patients with apparently acceptable adherence (>50%) likely reflects the limitations of dispensing-based adherence measures, which do not account for actual inhaler use, technique, or persistence. Furthermore, in severe asthma, symptoms may persist despite appropriate adherence due to refractory disease or suboptimal therapeutic response. As suggested by Tsao et al. [5], SABA overuse should not be interpreted solely as a consequence of poor adherence but rather as a multifactorial phenomenon involving treatment adequacy, disease phenotype, and patient behavior. These findings reinforce the importance of comprehensive assessment, including inhaler technique, treatment optimization, and phenotype-driven therapy.

Current international guidelines strongly discourage SABA-only treatment and recommend anti-inflammatory reliever strategies, such as inhaled corticosteroid–formoterol therapy, across all treatment steps [18,19]. Our findings provide real-world support for these recommendations, as persistent reliance on SABAs was common even in a population receiving high-intensity maintenance therapy. This suggests that implementation gaps remain and highlights the need for continued efforts to translate guideline recommendations into clinical practice.

Notably, patients receiving biologic therapies in our cohort exhibited a significantly lower prevalence of reliever overuse. Although causality cannot be established in this observational study, this finding is consistent with the known efficacy of biologics in reducing exacerbations, improving symptom control, and targeting underlying type 2 inflammation. Reduced reliance on rescue medication may therefore serve as a practical, real-world indicator of treatment response. This observation is particularly relevant given the increasing use of biologic therapies in severe asthma and supports their role not only in reducing exacerbations but also in improving day-to-day symptom burden.

The role of comorbidities should also be considered when interpreting reliever overuse. In our study, bronchiectasis was associated with the highest frequency of overuse, suggesting that coexisting airway diseases may contribute to persistent symptoms and increased bronchodilator use. This finding may reflect a higher symptom burden in these patients, including chronic cough, sputum production, and airflow limitation, which could drive a greater perceived need for short-acting bronchodilators. Additionally, bronchiectasis in the context of severe asthma might represent a distinct clinical phenotype characterized by persistent airway inflammation, mucus hypersecretion, and recurrent infections, all of which may contribute to poorer symptom control and increased reliever use. Alternatively, the frequent use of bronchodilators in this subgroup could be related to attempts to facilitate mucus clearance rather than to relieve bronchoconstriction alone. Further studies are warranted to better characterize this phenotype and to determine whether tailored therapeutic strategies may reduce rescue medication dependence in this population.

Similarly, other comorbid conditions such as gastroesophageal reflux, anxiety, and depression may influence symptom perception, treatment adherence, and healthcare-seeking behavior. These findings underscore the importance of a holistic approach to severe asthma management, including systematic evaluation and treatment of comorbidities.

Another important consideration is the behavioral component of reliever overuse. Patient perceptions of asthma control, reliance on rapid symptom relief, and potential misconceptions regarding the role of maintenance therapy may all contribute to excessive SABA use. Educational interventions targeting these factors may be critical to reducing overuse and improving outcomes. In addition, digital health tools and pharmacy-based monitoring strategies may offer opportunities for early identification of high-risk patients through tracking of inhaler dispensing patterns.

From a clinical perspective, our findings support the routine monitoring of rescue inhaler use as a simple and accessible tool for risk assessment in severe asthma. Unlike spirometry or biomarker measurements, inhaler use can be easily tracked through prescription data and may provide real-time insights into disease control. Incorporating this parameter into routine clinical practice could facilitate earlier intervention, treatment adjustment, and potentially reduce the risk of exacerbations and healthcare utilization.

This study has several limitations that should be acknowledged. Its retrospective and single-center design may limit generalizability, and the use of dispensing data as a proxy for medication use introduces potential misclassification. In addition, subgroup analyses were limited by sample size, and residual confounding cannot be excluded. Nevertheless, the study provides valuable real-world evidence in a population that is often underrepresented in clinical research and highlights clinically relevant patterns of rescue inhaler use.

Overall, our findings, together with the evidence from Tsao et al. [5] and prior SABINA studies, reinforce the clinical importance of SABA overuse as a marker of poor control and increased risk in asthma. In severe asthma, where disease burden is high and management is complex, reliever overuse should prompt careful reassessment of treatment, adherence, comorbidities, and disease phenotype. Future research should explore prospective strategies to reduce overuse and evaluate whether targeted interventions based on inhaler use patterns can improve long-term outcomes.

## 7. Conclusions

Rescue inhaler overuse is common in severe asthma and closely linked to poor symptom control, reflecting the combined effects of disease burden, treatment response, patient behavior, and comorbidities. Reduced overuse among biologic-treated patients supports its potential as a real-world marker of treatment effectiveness, and routine monitoring may help identify at-risk patients and guide individualized management.

## Figures and Tables

**Table 1:** Patient characteristics.

Characteristic	Total (N = 223)
Mean age (±SD) yr.	61.3 ± 15.04
Female	157 (70.4%)
Comorbidity associated with severe asthma	
Gastroesophageal reflux	79 (35.4%)
Bronchiectasis	77 (34.5%)
Allergic rhinitis	69 (30.9%)
Anxiety/Depression	31 (13.9%)
Chronic obstructive pulmonary disease	27 (12.1%)
Aspirin-exacerbated respiratory disease	26 (11.6%)
Allergic bronchopulmonary aspergillosis	13 (5.8%)
Vocal cord dysfunction	5 (2.2%)

**Table 2:** Reliever inhalers overuse.

Reliever Inhaler Prescribed (N = 144)	N (SABAs + SAMAs)	95% CI	SAMAs	SABAs
**Reliever inhaler overuse**	85/144 (59.0%)	50.9–66.7%	10/21 (47.6%)	75/123 (61.0%)
Low overuse (<three canisters withdrawals/four months)	68/144 (47.2%)	39.2–55.3%	8/21 (38.1%)	60/123 (48.8%)
High overuse (≥three canisters withdrawals/four months)	16/144 (11.1%)	7.0–17.3%	2/21 (9.5%)	14/123 (11.4%)

SABAs: short-acting β_2_-agonists; SAMAs: short-acting muscarinic antagonists.

**Table 3:** Multivariable Analysis.

Variable	Exp (B)	95% CI for Exp (B) (Lower–Upper)	*p*-Value
ACT ≥ 20	0.228	0.095–0.548	0.001
Constant	3.778	—	0.000

**Table 4:** Pulmonary function tests differences in overuse vs. non-overuse of rescue inhalers.

Pulmonary Function Tests	Rescue Overuse	Mean	±SD	*p*-Value
FEV_1_ (% predicted)	yes	69.7	19.3	0.088
	no	76.1	23.3	
FEV_1_ (mL)	yes	1918.3	784.8	0.23
	no	2128.5	876.4	
FVC (% predicted)	yes	82.8	17.1	0.503
	no	89.6	18.6	
FEV_1_/FVC (%)	yes	69.3	14.7	0.612
	no	66.6	12.6	
VC (mL)	yes	2825.2	947.8	0.478
	no	3159.0	1010.3	
FENO (ppb)	yes	33.2	38.1	0.185
	no	43.3	46.9	

FEV_1_: forced expiratory volume in 1 s; FVC: forced vital capacity; VC: vital capacity; FENO: fractional exhaled nitric oxide.

**Table 5:** Rescue inhalers overuse according to asthma-related comorbidity.

Comorbidity Associated with Asthma	N	Rescue Inhaler Overuse	% Rescue Inhaler Overuse	Acceptable Adherence to Inhaled Maintenance Therapy (>50%)
Bronchiectasis	77	30/77	38.9%	63/77 (81.8%)
Anxiety/Depression	31	5/31	16.1%	25/31 (80.6%)
Allergic bronchopulmonary aspergillosis	13	1/13	7.7%	13/13 (100.0%)
COPD	27	2/27	7.4%	24/27 (88.9%)
Gastroesophageal reflux	79	5/79	6.3%	64/79 (81.0%)
Vocal cords dysfunction	5	0/5	0.0%	4/5 (80.0%)

## Data Availability

The original contributions presented in this study are included in the article. Further inquiries can be directed to the corresponding author.
